# Case Report: Pheochromocytoma in a 59-Year-Old Woman Presenting With Hypotension

**DOI:** 10.3389/fcvm.2021.648725

**Published:** 2021-03-11

**Authors:** Hao-Yu Wu, Tian-Jiao Gao, Yi-Wei Cao, Lei Liang

**Affiliations:** ^1^Department of Cardiology, Shaanxi Provincial People's Hospital, Xi'an, China; ^2^Department of Gastroenterology, Xi'an Children's Hospital, Xi'an, China; ^3^Department of Electrocardiology, Shaanxi Provincial People's Hospital, Xi'an, China

**Keywords:** pheochromocytoma, hypotension, shock, electrocardiogram, cardiovascular complication

## Abstract

**Background:** Pheochromocytoma patients who present with shock are extremely rare. Here, we report a patient who presented with shock and was diagnosed with pheochromocytoma.

**Case Summary:** A 59-year-old woman with a history of hypertension without any treatment for 5 years presented with chest tightness. Vital signs on arrival indicated blood pressure of 78/50 mmHg. Twelve-lead electrocardiogram indicated ST-segment depression in leads II, III, aVF, and V3–V6 and QT prolongation. Coronary angiogram revealed no evidence of coronary artery disease. Contrast-enhanced computed tomography demonstrated an inhomogeneous right adrenal mass (2.5 × 3.0 cm). Her 24-h urinary norepinephrine and catecholamine levels were elevated. The patient underwent laparoscopic right adrenalectomy. Histopathology confirmed adrenal pheochromocytoma with residual necrosis. The patient was diagnosed with pheochromocytoma. During the 2-year follow-up, the patient was asymptomatic, and her blood pressure remained normal without medication. ECG showed that the ST-segment depression in leads II, III, aVF, and V3–V6 and the QT prolongation had disappeared. The patient showed no signs of recurrence, with normal urine norepinephrine and catecholamine levels.

**Conclusion:** Patients with pheochromocytoma can present with hypotension or even shock. Clinicians should suspect pheochromocytoma when a patient with a history of hypertension has sudden hypotension or even shock.

## Introduction

Pheochromocytoma is a rare neuroendocrine tumor that originates from the adrenal medulla or extra-adrenal paraganglion chromaffin tissue and secretes catecholamines (1). The clinical manifestations of patients with pheochromocytoma are diverse, ranging from asymptomatic to cardiac arrest. The typical triad, including episodic headache, palpitations and sweating, only occurs in 24% of pheochromocytoma patients (2, 3). This often misleads clinicians to make a wrong diagnosis. Hypertension is one of the most common manifestations of pheochromocytoma and can be persistent or paroxysmal.

Shock is defined as insufficient perfusion of organs and peripheral tissues, and is classified as hypovolemic, cardiogenic, or restrictive (vasodilatation/distribution) according to its etiology. However, pheochromocytoma patients who present with shock are extremely rare. The pathophysiological factors of hypotension or shock include tumor necrosis leading to a sudden decrease in continuous catecholamine secretion, adrenergic receptor desensitization, and decreased vascular volume. Here, we report a case of a patient with pheochromocytoma characterized by shock.

## Case Presentation

A 59-year-old woman presented with chest tightness for 2 h. 2 h before admission, the patient experienced chest tightness accompanied by palpitations, dizziness, vomiting and sweating.

The patient had a history of hypertension for 5 years without any treatment or etiological diagnosis. The patient denied a family history of premature coronary artery disease and special personal history, such as smoking and drinking.

Vital signs on arrival indicated blood pressure 78/50 mmHg, heart rate 102 beats per min and a respiratory rate of 26 beats per minute. The patient was conscious, but her lips were cyanotic. Her face was pale, and her extremities were wet and cold. There was no obvious abnormality in the heart or lung examination. Jugular vein engorgement and peripheral edema were not found.

**Figure 1:**
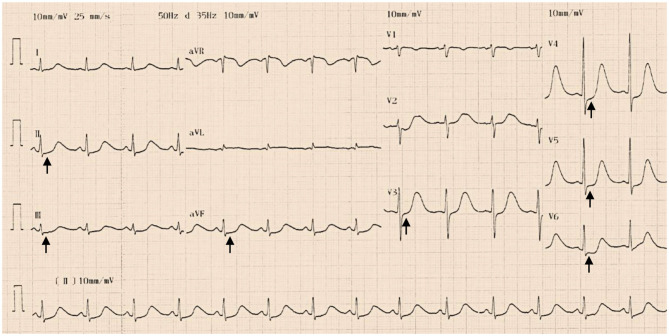
Twelve-lead electrocardiogram indicated a sinus rhythm with ST-segment depression in leads II, III, aVF, and V3–V6 (black arrows) and QT prolongation (QTc 529 ms) at admission.

**Figure 2:**
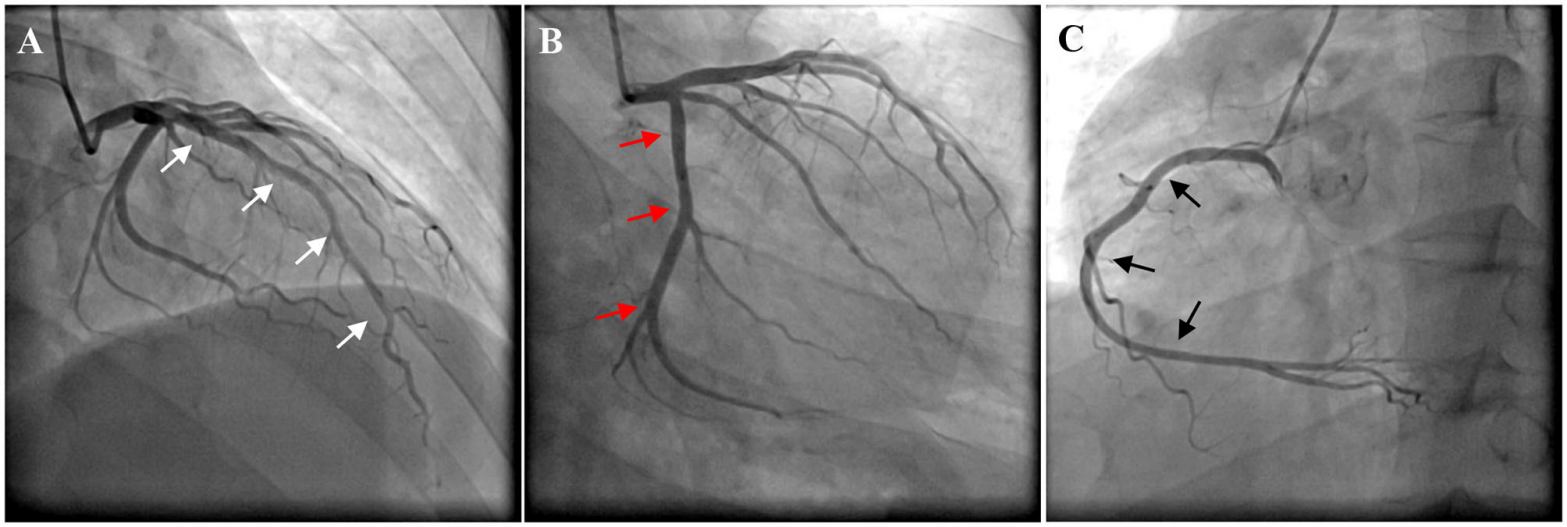
Coronary angiogram revealed no evidence of coronary artery disease. **(A)** Left anterior descending artery (white arrows). **(B)** Left circumflex artery (red arrows). **(C)** Right coronary artery (black arrows).

**Figure 3:**
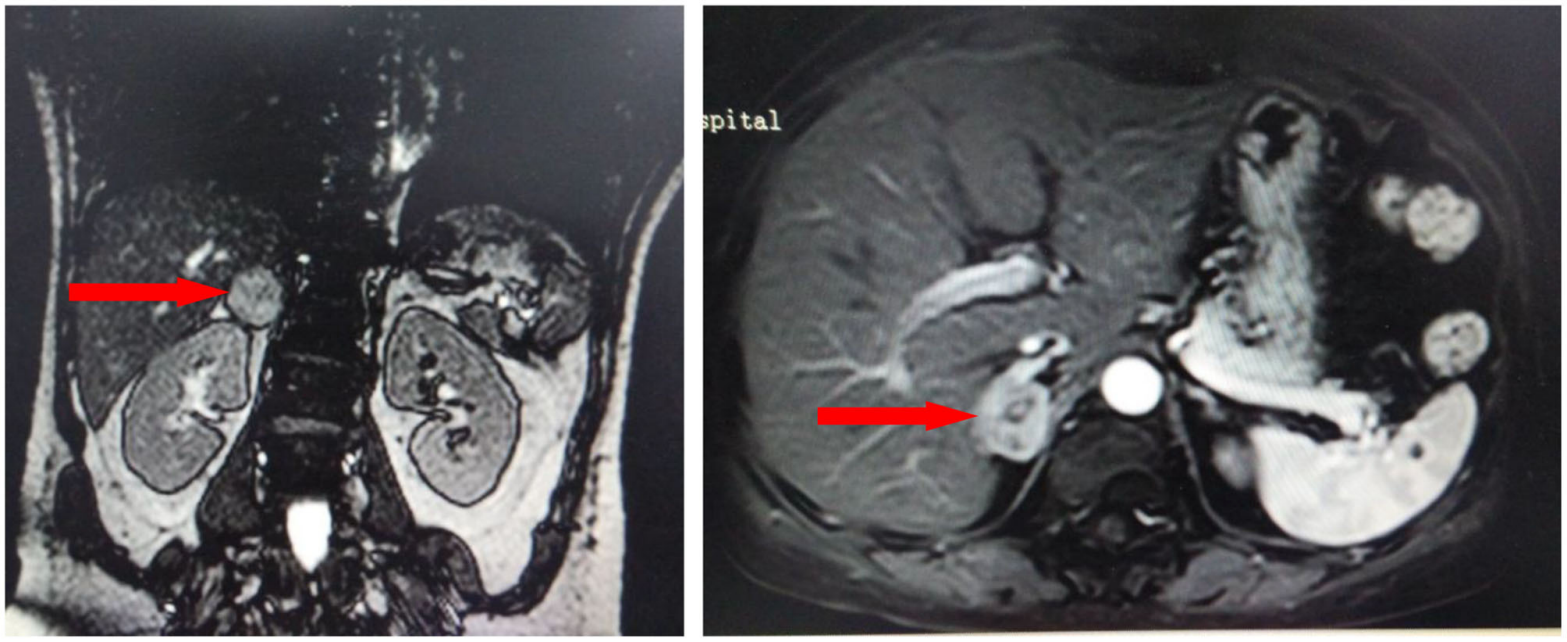
Contrast-enhanced computed tomography demonstrated an inhomogeneous right adrenal mass (2.5 × 3.0 cm, red arrows).

The troponin I level was 1.16 ng/mL (reference interval <0.04). Hemoglobin, leukocytes, electrolytes, B-type natriuretic peptide, liver function, renal function, and D-dimer were not significantly abnormal. Twelve-lead electrocardiogram (ECG) indicated a sinus rhythm with ST-segment depression in leads II, III, aVF, and V3–V6 and QT prolongation (QTc 529 ms) (Figure 1). Coronary angiogram revealed no evidence of coronary artery disease (Figure 2). Echocardiography showed a thickened ventricular septum (12 mm) and normal left ventricular function without abnormal wall motion (left ventricular ejection fraction of 55%). Chest computed tomography showed no obvious abnormality, but abdominal computed tomography showed an adrenal mass. Contrast-enhanced computed tomography demonstrated an inhomogeneous right adrenal mass (2.5 × 3.0 cm, Figure 3). The urinary norepinephrine level was 288.8 nmol/24 h (reference interval 80.3–164.0), and the urinary catecholamine level was 307.4 nmol/24 h (reference interval 94.5–238.3). 24 h urinary epinephrine level was normal.

Saline solution and dopamine injection were administered to maintain blood pressure. The patient's condition gradually improved, and her blood pressure gradually stabilized. 7 days later, the patient was transferred to the urology department and successfully underwent laparoscopic right adrenalectomy. Histopathology confirmed adrenal pheochromocytoma with residual necrosis. Immunohistochemistry confirmed that chromogranin A, neuron-specific enolase and synaptophysin were positive. The patient was diagnosed with pheochromocytoma.

**Figure 4:**
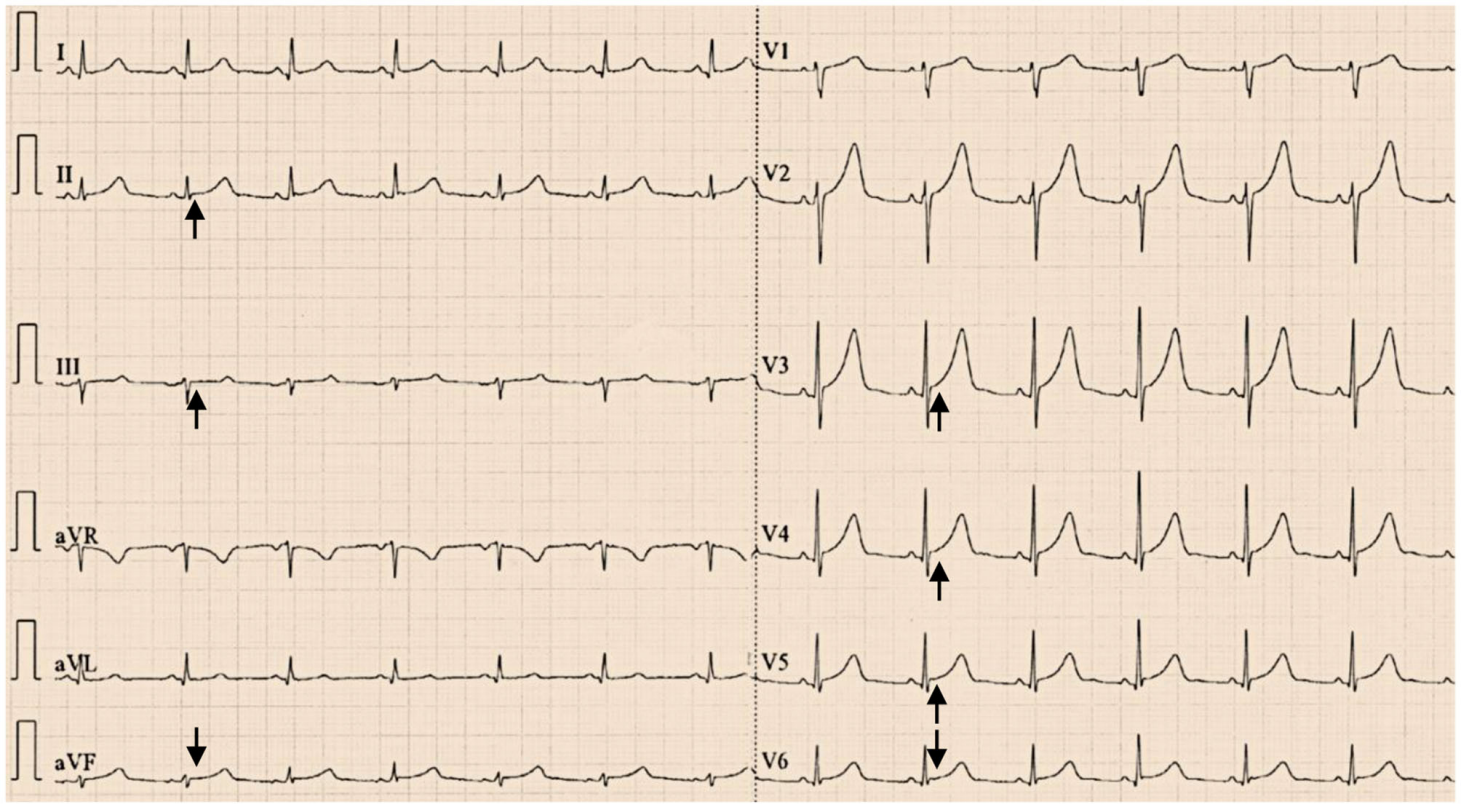
Twelve-lead electrocardiograms showed that the ST-segment depression in leads II, III, aVF, and V3–V6 had disappeared (black arrows) with normal QT interval (QTc 424 ms) at the 2-year follow-up after resection of the pheochromocytoma.

The patient was free of complications during hospitalization. During the 2-year follow-up, the patient was asymptomatic, and her blood pressure remained normal without medication. ECG showed that the ST-segment depression in leads II, III, aVF, and V3–V6 and the QT prolongation had disappeared (Figure 4). The patient showed no signs of recurrence, with normal urine norepinephrine and catecholamine levels (Table 1).

**Table 1:** Timeline table with relevant laboratory data from the episode of care.

	**Patient's value**	**Reference interval**
**Initial laboratory values on presentation**		
Hemoglobin, g/L	124.0	113.0–151.0
Leukocytes, ×10^9^/L	5.6	3.7–9.2
Troponin I, ng/mL	1.16	<0.04
B-type natriuretic peptide, pg/ml	58.0	<76.0
D-dimer, mg/L	0.2	<0.6
Serum sodium, mmol/L	3.9	3.5–5.5
Serum potassium, mmol/L	141.0	135.0–148.0
Creatinine, μmol/L	45.4	45.0–105.0
Alanine aminotransferase, U/L	38.0	5.0–40.0
Glutamic oxaloacetic transaminase, U/L	34.0	5.0–40.0
Urinary norepinephrine, nmol/24 h	288.8	80.3–164.0
Urinary catecholamine, nmol/24 h	307.4	94.5–238.3
Urinary epinephrine, nmol/24 h	18.6	12.5–70.4
**Laboratory values at the 2-year follow-up**		
Urinary norepinephrine, nmol/24 h	128.2	80.3–164.0
Urinary catecholamine, nmol/24 h	196.3	94.5–238.3
Urinary epinephrine, nmol/24 h	21.4	12.5–70.4

## Discussion

Pheochromocytoma can produce excessive amounts of catecholamines, especially epinephrine and norepinephrine, and release them continuously or intermittently (4, 5). Pheochromocytoma has various clinical manifestations. The typical triad of pheochromocytoma, including episodical headache, palpitations and sweating, lasts from a few minutes to a few hours as a direct consequence of excessive catecholamine secretion and is often accompanied by hypertension (6). Approximately 90% of patients with pheochromocytoma present with sustained or paroxysmal hypertension (7, 8). Hypertension in pheochromocytoma usually manifests as high peripheral resistance and low heart index. Norepinephrine secreted by pheochromocytoma increases peripheral vascular resistance, leading to increased systolic and diastolic blood pressure (9). Epinephrine-secreting pheochromocytoma usually causes patients to experience paroxysmal symptoms, such as headaches, palpitations, sweating and anxiety, while patients with norepinephrine-secreting tumors usually have persistent symptoms (such as persistent hypertension) related to the continuous catecholamine overdose (10). In our case, the patient's norepinephrine level was elevated, but the epinephrine level was normal. The patient did not present with the typical triad of pheochromocytoma, which may be because the tumor secreted norepinephrine rather than epinephrine. The patient had a history of hypertension for 5 years, and persistent hypertension is related to the continuous catecholamine overdose produced by norepinephrine-secreting tumors.

Occasionally, patients with pheochromocytoma experience hypotension or even shock. The pathophysiological factors of hypotension and shock include tumor necrosis leading to a sudden decrease in continuous catecholamine secretion, adrenergic receptor desensitization, and decreased vascular volume (11). Some cardiovascular events, such as myocardial infarction and arrhythmia, can also cause shock (12). In our case, the pheochromocytoma concomitant with circulatory shock may have been related to the sudden decrease in catecholamine secretion caused by tumor necrosis, and histopathology confirmed an adrenal pheochromocytoma with residual necrosis.

Pheochromocytoma can also cause other cardiovascular complications, including cardiac hypertrophy, heart failure, arrhythmias, ischemic heart disease and even acute myocardial infarction, which are due to the effects of secreted catecholamines (3, 13). Norepinephrine secreted by pheochromocytoma can cause structural and functional remodeling of the heart, such as left ventricular hypertrophy. Catecholamines, especially norepinephrine, can cause myocardial damage by increasing the oxygen consumption and apoptosis of cardiomyocytes and can further lead to left ventricular systolic dysfunction and dilated cardiomyopathy (14, 15). Some factors can lead to an imbalance of the oxygen supply and demand, such as cardiotoxicity of catecholamines, increased muscle mass and coronary artery spasm, causing myocardial ischemic necrosis (16, 17). Patients may have chest pain and tightness, and ECG may manifest as ST-segment elevation or depression. Myocardial enzymes may be elevated (18, 19). In some cases, pheochromocytoma is associated with Takotsubo cardiomyopathy, characterized by reversible left ventricular apical ballooning (20–22). The level of B-type natriuretic peptide is generally significantly elevated in these patients (23). In our case, the typical wall motion abnormalities of Takotsubo cardiomyopathy were not reflected in echocardiography, and the B-type natriuretic peptide level was normal. Therefore, the suspicion of Takotsubo cardiomyopathy caused by pheochromocytoma was not confirmed. The ECG of patients with pheochromocytoma may also show a variety of abnormalities in the heart rhythm, conduction, and repolarization, including a significantly prolonged QT interval and deep and wide, symmetrical, inverted T waves. A prolonged QT interval may induce the risk of torsade de pointes ventricular tachycardia (24). In our case, preoperative ECG showed ST-segment depression in leads II, III, aVF and V3–V6 and QT prolongation, and follow-up ECG showed that these changes had disappeared after laparoscopic adrenalectomy.

## Limitations

The patient did not undergo echocardiography again to determine whether the ventricular septum returned to normal thickness during the 2-year follow-up.

## Conclusion

Occasionally, patients with pheochromocytoma can present with hypotension or even shock. Pheochromocytoma should be suspected when a patient with a history of hypertension has sudden hypotension or even shock.

## Data Availability Statement

The raw data supporting the conclusions of this article will be made available by the authors, without undue reservation.

## Ethics Statement

Written informed consent was obtained from the individual(s) for the publication of any potentially identifiable images or data included in this article.

## Author Contributions

All authors contributed in this patient care, diagnosis and treatment, and in writing this article, contributed to the article, and approved the submitted version.

## Conflict of Interest

The authors declare that the research was conducted in the absence of any commercial or financial relationships that could be construed as a potential conflict of interest.
